# Supplementary material for: Effects of Xanthine Oxidase Inhibition by Febuxostat on Lipid Profiles of Patients with Hyperuricemia: Insights from Randomized PRIZE Study
Source: Nutrients. 2024 Jul 19;16(14):2324. doi: 10.3390/nu16142324 (PMC11280470; doi:10.3390/nu16142324)
Supplement: Supplementary file 1 [file nutrients-16-02324-s001.zip › nutrients-3107496-supplementary.pdf]

Figure S1.

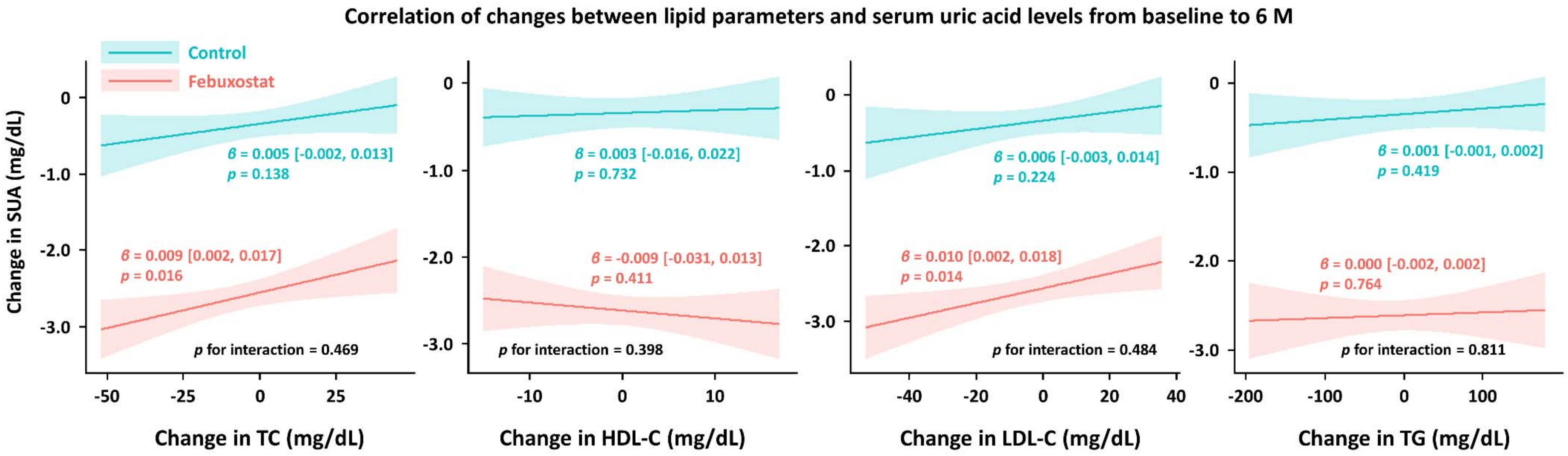

Table S1. Changes from baseline in cardiometabolic variables at 24 months.

Variable	Febuxostat Group Changes (95% CI)	p-Value (for Changes)	Control group Changes (95% CI)	p-Value (for Changes)	Group Difference (95% CI)	p-Value (for Groups)
Non-HDL-C (mg/dL)	-7.2 (-10.4 to -4.1)	<0.001	-4.4 (-7.6 to -1.1)	0.005	-2.9 (-7.4 to 1.7)	0.214
Total cholesterol (mg/dL)	-6.2 (-9.4 to -2.9)	<0.001	-4.2 (-7.6 to -0.9)	0.010	-1.9 (-6.6 to 2.8)	0.420
HDL-C (mg/dL)	1.7 (0.4 to 3.0)	0.049	0.7 (-0.6 to 2.0)	0.668	1.0 (-0.8 to 2.8)	0.294
LDL-C (mg/dL)	-3.9 (-6.9 to -1.0)	0.002	-3.8 (-6.9 to -0.7)	0.005	-0.2 (-4.4 to 4.1)	0.944
Triglyceride (mg/dL)	1.9 (-9.3 to 13.1)	0.079	10.6 (-1.0 to 22.1)	0.762	-8.7 (-24.6 to 7.2)	0.283
Serum uric acid (mg/dL)	-3.0 (-3.1 to -2.8)	<0.001	-0.3 (-0.5 to -0.2)	<0.001	-2.6 (-2.9 to -2.4)	<0.001
Body mass index (kg/m^2)	-0.0 (-0.2 to 0.1)	0.502	-0.2 (-0.4 to 0.0)	0.045	0.1 (-0.1 to 0.4)	0.325
Systolic BP (mmHg)	-0.7 (-2.7 to 1.2)	0.567	0.3 (-1.8 to 2.3)	0.233	-1.0 (-3.8 to 1.9)	0.496
eGFR (mL/min/1.73 m^2)	-2.0 (-3.0 to -1.0)	<0.001	-1.8 (-2.8 to -0.8)	<0.001	-0.2 (-1.6 to 1.2)	0.778

CI, confidence interval; BP, blood pressure; eGFR, estimated glomerular filtration rate; HDL-C, high-density lipoprotein cholesterol; LDL-C, low-density lipoprotein cholesterol; TG, triglyceride.
